# Crystal methamphetamine use in British Columbia, Canada: A cross-sectional study of people who access harm reduction services

**DOI:** 10.1371/journal.pone.0252090

**Published:** 2021-05-26

**Authors:** Kristi Papamihali, Dylan Collins, Mohammad Karamouzian, Roy Purssell, Brittany Graham, Jane Buxton

**Affiliations:** 1 British Columbia Centre for Disease Control, Vancouver, British Columbia, Canada; 2 Faculty of Medicine, University of British Columbia, Vancouver, British Columbia, Canada; 3 School of Population and Public Health, Faculty of Medicine, University of British Columbia, Vancouver, British Columbia, Canada; 4 HIV/STI Surveillance Research Center, and WHO Collaborating Center for HIV Surveillance, Institute for Futures Studies in Health, Kerman University of Medical Sciences, Kerman, Iran; 5 Department of Emergency Medicine, Faculty of Medicine, University of British Columbia, Vancouver, British Columbia, Canada; 6 British Columbia Drug and Poison Information Centre, Vancouver, British Columbia, Canada; University of Toronto, CANADA

## Abstract

**Introduction:**

Increased use of crystal methamphetamine (“crystal meth”) has been observed across North America and international jurisdictions, including a notable increase in the presence of methamphetamines in illicit drug toxicity deaths in British Columbia (BC), Canada. We used data from a cross-sectional survey and urine toxicology screening to report the prevalence, correlates, and validity of self-reported crystal meth use among clients of harm reduction sites in BC.

**Materials and methods:**

Survey data were collected from 1,107 participants across 25 communities in BC, through the 2018 and 2019 Harm Reduction Client Survey. We described reported substance use and used a multivariate logistic regression model to characterize crystal meth use. Urine samples provided by a subset of participants were used to derive validity of self-reported three-day crystal meth use compared to urine toxicology screening.

**Results:**

Excluding tobacco, crystal meth was the most frequently reported substance used in the past three days in 2018 and 2019 (59.7% and 71.7%, respectively). Smoking was the dominant route of administration for crystal meth, crack, heroin, and fentanyl. Multivariate analysis determined significantly higher odds of crystal meth use among those who used opioids (Adjusted Odds Ratio [AOR] = 3.13), cannabis (AOR = 2.10), and alcohol (1.41), and among those who were not regularly housed (AOR = 2.08) and unemployed (AOR = 1.75). Age ≥50 was inversely associated with crystal meth use (AOR = 0.63). Sensitivity of self-reported crystal meth use was 86%, specificity was 86%, positive predictive value was 96%, and negative predictive value was 65%.

**Conclusions:**

Crystal meth was the most commonly used substance among clients of harm reduction sites in BC in 2018 and 2019, and was frequently used concurrently with opioids. Comparison to urine samples demonstrated high validity of self-reported crystal meth use. Understanding evolving patterns of substance use will be imperative in tailoring harm reduction and substance use services for individuals that use crystal meth.

## Introduction

British Columbia (BC), the westernmost province of Canada, is an epicentre of the opioid overdose epidemic that has swept North America, primarily driven by fentanyl [1]. The public health focus on opioids, although essential, has potentially masked a surge of crystal methamphetamine use that has been noted internationally. In particular, reports from the United States (US) and Canada demonstrate an increase in co-use of crystal methamphetamine with opioids [2–4]. This, coupled with underdeveloped harm reduction and treatment strategies for methamphetamine use disorders, has created a need for epidemiological studies of crystal methamphetamine (“crystal meth”) use in BC.

Crystal meth is an illegal, highly addictive, synthetic central nervous system stimulant used for a variety of recreational (e.g., euphoria, enhanced sexual performance) and functional (e.g., increased alertness, productivity, vigilance, coping) purposes [5, 6]. According to the 2019 World Drug Report, amphetamines and prescription stimulants are the third most commonly used drug worldwide (following cannabis and opioids) with the highest prevalence of use in North America [7]. Across Canada, the US, and Australia, there has been an increased use of crystal meth reflected in notable increases in the presence of methamphetamines in illicit drug toxicity deaths, methamphetamines seizures by law enforcement, and methamphetamine-related hospitalizations [7, 8].

Crystal meth attributable morbidity and mortality include fatal overdoses, profound weight loss, psychosis, neurotoxicity, cognitive impairment, cardiovascular pathology, and an increased risk of contracting HIV and other sexually transmitted infections through unprotected sexual contact and syringe sharing [3, 9–13]. Moreover, it is deeply interconnected with non-fatal overdoses, transition to injection drug use, and further social marginalization [14–17]. Although usually smoked by new users, crystal meth use by injection-naïve youth is associated with the subsequent initiation of injection drug use [16, 18]. In BC, concomitant use of crystal meth amongst people who use more than one substance is also the strongest predictor of non-fatal overdoses [15]. Crystal meth’s use is closely tied to social circumstances, including stable housing; eviction amongst people who use substances is independently associated with the initiation or relapse of crystal meth use, in part to cope, to maintain alertness to protect oneself and their belongings, and to suppress appetite [5, 19].

Crystal meth use needs to be understood both within the context of opioid use and outside of opioid use, considering the harmful consequences that may arise. Much of the existing literature from BC has focused on highly selective cohorts in Downtown Vancouver [17, 20], and the global literature is limited in that drug use patterns vary widely and are not necessarily generalizable to areas dealing with epidemics of fentanyl-related overdoses. The aim of this study was to build on previous work to describe the epidemiology of crystal meth use among people who use substances in BC. To achieve this aim, the objectives of the study were to: (i) estimate the prevalence of crystal meth use amongst people who access harm reduction services; (ii) characterize the socio-demographic and individual-level determinants of crystal meth use among people who use crystal meth; and (iii) determine the accuracy of self-reported crystal meth use in comparison with urine toxicology screening.

## Materials and methods

### Data source

Data for this study was collected through two iterations of the BC Harm Reduction Client Survey (HRCS), administered in 2018 (May to August) and 2019 (October to December). The HRCS is a cross-sectional survey of people who use substances administered at harm reduction supply distribution sites in BC that aims to collect information on substance use patterns, associated harms, stigma, and utilization of harm reduction services. Findings of the survey are then used to inform provincial harm reduction planning and evaluate service quality, in addition to investigating emerging substance use trends. The survey was originally piloted and evaluated in 2012 [21] and administered each year, 2012 to 2015. Data collection methods for previous iterations as well as the 2018 iteration have been previously described in detail elsewhere [21–23].

For 2018 and 2019 iterations, the survey instrument was adapted to reflect current needs and input from stakeholders. Survey questions were reviewed by the research team, harm reduction program coordinators, and people with lived and living experience of substance use to assess validity and relevance. Additionally, the surveys were piloted each year with people who use substances attending services at Vancouver Area Network of Drug Users (VANDU), a community organization providing harm reduction services, education, and other resources for people who use substances in Vancouver, BC. Survey instruments are available in S1 Appendix for 2018 and S2 Appendix for 2019.

Participating sites were selected from a network of sites that distribute supplies for safer sex and substance use through the BC Centre for Disease Control (BCCDC) Harm Reduction Program. Sites offer a variety of services in addition to harm reduction supply distribution, including but not limited to overdose prevention and response training, supervised consumption services, and peer support programs for people who use substances and/or people living with HIV.

We employed a two-stage convenience sampling approach to identify participating sites. Regional harm reduction program coordinators helped identify sites suitable for participation in each of the five health regions in BC. Sites were then consulted and recruited based on willingness to participate and logistical capacity. Trained site staff and volunteers were responsible for recruitment of participants and survey administration. Sites were given two weeks for completion of data collection.

Eligibility criteria for survey participation included age of 19 years or older, self-reported substance use of any substances other than or in addition to cannabis in the past six months, and ability to provide verbal informed consent. The interviewer-administered, paper-based questionnaire was four pages long and took approximately 10 minutes to complete. Survey questions explored substance patterns and harm reduction service use, including past three-day substance use practices and experiencing a drug overdose. Participants were provided $5 CAD for survey participation and an additional $5 CAD was paid to participants who also provided a urine sample. Additionally, sites received $5 CAD for each participant surveyed to compensate for any costs incurred due to data collection. Surveys were mailed to the BCCDC for data entry and analysis.

### Urine toxicology screening

A subset of sites that participated in survey administration also participated in collection of urine samples from participants. Detailed methods have been previously described [22]. In 2018, participation in urine sample collection was voluntary and was based on site capacity and logistics (e.g., availability of a washroom on-site). In 2019, all sites were required to collect urine samples in order to participate in the survey. However, if site capacity or ability to collect urine samples changed following study recruitment in 2019, sites were still encouraged to participate in survey data collection. Similarly, if participants decided not to provide a urine sample after completing the survey, site staff were instructed to retain survey data, send to BCCDC, and compensate participants for survey participation.

Urine samples were sent to a Life Labs laboratory based in Toronto, Ontario (www.lifelabs.com) for a Broad-Spectrum Urine Toxicology Screen. The urine toxicology screen assessed for the presence of a variety of substances and their metabolites, including opioids, amphetamines, benzodiazepines, anti-depressants, anti-psychotics, cannabinoids, and other substances. Presence of substances was confirmed through liquid chromatography and mass spectrometry, based on Life Labs detection limits for each substance (available upon request). Urine toxicology screen data was linked with survey data based on unique and anonymous identifiers assigned to each participant.

### Study variables

The primary outcome variable in this study was past three-day self-reported crystal meth use. Information on route of administration and other substances used in the past three days was also reported, including use of crack, cocaine, other stimulants, heroin, fentanyl, methadone, morphine, oxycodone, hydromorphone (Dilaudid), benzodiazepines, alcohol, cannabis, and tobacco. Buprenorphine/naloxone (Suboxone) was added to the list of substances in 2019. A variable denoting polysubstance use was also created referring to use of any opioids and stimulants, opioids and benzodiazepines, or stimulants and benzodiazepines in the past three days. Other variables related to substance use included experiencing an opioid overdose in the past six months and experiencing a stimulant overdose in the past six months.

Demographic variables included regional health authority and urbanicity of survey administration sites, gender, age, employment status, and housing status (being not currently regularly housed was defined as currently being homeless, having no regular place to stay, having no fixed address, couch surfing, or living in a shelter). Urbanicity of sites was derived using a classification system developed by the BC Ministry of Health specific to communities in BC, which combined definitions of urbanicity set by Statistics Canada with indicators of remoteness, population density, and proximity to urban areas [24, 25].

### Data analysis

We described past three-day substance use and route of administration for each substance reported for all survey participants in 2018 and 2019. To characterize recent crystal meth use, an analytic sample with complete responses for all substance use and demographic variables was derived. Responses where participants indicated “prefer not to say” were treated as missing data and excluded from further analysis. Frequency distributions and bivariate analyses with chi-square tests of independence or Fisher’s exact test where appropriate were conducted for all variables to describe characteristics of participants that reported crystal meth use.

We constructed a multivariate logistic regression model to identify factors associated with crystal meth use. Bivariate logistic regression analyses of independent variables with the outcome variable were conducted, and variables with a *P*-value less than 0.25 were included in further modeling [26, 27]. Participants that identified as transgender or gender-expansive (n = 17) were excluded from multivariate analysis due to small cell sizes. Additionally, the variable denoting polysubstance use in the past three days was not included in the model building process, given that it was derived from both the explanatory and outcome variables. Variables included in the final multivariate model were selected through a backward selection approach and final model fit was assessed using Akaike’s Information Criterion [28]. Odds ratios (OR), adjusted odds ratios (AOR) and 95% confidence intervals (95% CI) were derived from the final multivariate logistic regression model. To verify findings from complete case analysis, estimates from a parallel model were derived using all available data, including data from participants that had missing responses or indicated “prefer not to say” for independent variables. For the purposes of this verification, missing and “prefer not to say” responses were grouped into an “unknown” level for each variable. All statistical analyses were conducted using R version 3.6.1 [29].

### Measures of validity

A secondary component of this study was to determine validity of self-reported crystal meth use when compared with urine toxicology screen. We compared past three-day self-reported crystal meth use with detection of methamphetamine in urine toxicology screening for the subset of participants that participated in urine screening. Past three-day self-report allows for comparison with substances detected in urine as methamphetamine and metabolites can be detected in urine samples between 1 to 3 days following use [30]. Six measures of validity [31] for self-reported crystal meth use along with 90% confidence intervals were calculated and defined as follows:

- *Sensitivity*: The probability of reporting crystal meth use among participants that had methamphetamine detected in a urine toxicology screen
- *Specificity*: The probability of not reporting crystal meth use among participants that did not have methamphetamine detected in a urine toxicology screen
- *Positive predictive value (PPV)*: The probability of detecting methamphetamine in a urine toxicology screen among participants that reported using crystal meth
- *Negative predictive value (NPV)*: The probability of not detecting methamphetamine in a urine toxicology screen among participants that did not report using crystal meth
- *Positive likelihood ratio*: The probability of reporting crystal meth use when methamphetamine is detected compared to the probability reporting crystal meth use when methamphetamine is not detected
- *Negative likelihood ratio*: The probability of not reporting crystal meth use when methamphetamine is detected compared to the probability not reporting crystal meth use when methamphetamine is not detected

### Ethical considerations

The current study was reviewed and approved by the University of British Columbia Office of Behavioural Research Ethics (#H07-00570). Verbal informed consent was sought by site staff for both survey and urine toxicology screen participation. Site staff reviewed study information and protocols with participants prior to beginning data and urine specimen collection. Participants were asked to verbally consent to the process and were reminded that they may skip questions or end early without any consequences. Participants’ refusal for participation in the study did not impact their receipt of services at the site in any way.

## Results

### Study sample characteristics

A total of 1,107 participants participated in the 2018 and 2019 HRCS, with 486 participants in 2018 and 621 participants in 2019. In 2018, a total of 37 sites were approached for participation, of which 27 sites from 22 communities had the capacity to participate. In 2019, 43 sites were approached, of which 22 sites from 20 communities had the capacity to participate. Across the two survey cycles, there was representation from 32 different harm reduction supply distribution sites from 25 communities across BC, with 17 sites and 17 communities that participated in both the 2018 and the 2019 cycle. Overall, 23 sites were located in medium and large urban areas, and 9 in small urban and rural communities.

Table 1 presents frequencies of past three-day substance use and route of administration among participants. Crystal meth was the most commonly reported substance used in the past three days in 2018 (59.7%) and 2019 (71.7%), not including tobacco. Most participants reported smoking crystal meth– 67.2% in 2018 and 79.6% in 2019 –while one-third reported injecting each year. Not considering cannabis, alcohol, and tobacco, other substances commonly reported included heroin, fentanyl, methadone, crack, and cocaine. Reported use of these substances was similar across both study years. The median number of substances reported used in the past three days (including cannabis and alcohol) was 3 (IQR: 2–5), and polysubstance use involving opioids, stimulants, and/or benzodiazepines was reported by 46.7% of all participants.

**Table 1:** Self-reported past three-day substance use among 2018 and 2019 Harm Reduction Client Survey (HRCS) participants (N = 1,107).

Substance	2018 (N = 486)				2019 (N = 621)			
	Total^a^	Route of administration^b^			Total^a^	Route of administration^b^		
		Smoking	Injection	Other		Smoking	Injection	Other
	n (%)	n (%)	n (%)	n (%)	n (%)	n (%)	n (%)	n (%)
**Opioids**								
Heroin	212 (43.6%)	125 (59.0%)	113 (53.3%)	10 (4.7%)	272 (43.8%)	200 (73.5%)	135 (49.6%)	21 (7.7%)
Fentanyl	188 (38.7%)	106 (56.4%)	96 (51.1%)	10 (5.3%)	283 (45.6%)	190 (67.1%)	135 (47.7%)	18 (9.5%)
Methadone	133 (27.4%)	13 (9.8%)	17 (12.8%)	89 (66.9%)	139 (22.4%)	17 (12.2%)	4 (2.9%)	102 (73.4%)
Morphine	59 (12.1%)	9 (15.3%)	34 (57.6%)	15 (25.4%)	70 (11.3%)	18 (25.7%)	26 (37.1%)	31 (44.3%)
Oxycodone	23 (4.7%)	2 (8.7%)	9 (39.1%)	9 (39.1%)	6 (1.0%)	2 (33.3%)	3 (50.0%)	5 (83.3%)
Hydromorphone (Dilaudid)	25 (5.1%)	3 (12.0%)	12 (48.0%)	8 (32.0%)	17 (2.7%)	4 (23.5%)	4 (23.5%)	10 (58.8%)
Buprenorphine/ naloxone (Suboxone)^c^	-	-	-	-	25 (4.0%)	2 (8.0%)	1 (4.0%)	17 (68.0%)
**Stimulants**								
Crystal meth	290 (59.7%)	195 (67.2%)	104 (35.9%)	28 (9.7%)	445 (71.7%)	354 (79.6%)	143 (32.1%)	74 (16.6%)
Crack	102 (21.0%)	80 (78.4%)	10 (9.8%)	6 (5.9%)	141 (22.7%)	127 (90.1%)	4 (2.8%)	6 (4.2%)
Cocaine (powder)	88 (18.1%)	41 (46.6%)	20 (22,7%)	30 (34.1%)	105 (16.9%)	48 (45.7%)	22 (20.9%)	53 (50.5%)
Other stimulants^d^	26 (5.3%)	6 (23.1%)	6 (23.1%)	8 (30.8%)	42 (6.8%)	11 (26.2%)	13 (31.0%)	22 (52.4%)
**Other substances**								
Benzodiazepines	53 (10.9%)	4 (7.5%)	5 (9.4%)	33 (62.3%)	70 (11.3%)	12 (17.1%)	12 (17.1%)	41 (58.6%)
Alcohol	172 (35.4%)	-	-	-	229 (36.9%)	-	-	-
Cannabis/hash	234 (48.1%)	-	-	-	328 (52.8%)	-	-	-
Tobacco	338 (69.5%)	-	-	-	516 (83.1%)	-	-	-

^a^ Participants could report use of more than one substance; thus, column percentages may add up to more than 100%.

^b^ Participants could indicate more than one route of administration if applicable. Row percentages presented for route of administration.

^c^ Data on buprenorphine/naloxone (Suboxone) use was not available from the 2018 survey.

^d^ Other stimulants may include Ritalin, Adderall, or other stimulants participants may have identified as a stimulant.

Among participants, 36.9% reported injecting at least one substance, 64.5% reported smoking at least one substance (other than cannabis and tobacco), and 27.8% reported both smoking and injecting at least one substance in the past three days. Smoking was more frequently reported as the route of administration in 2019 when compared to 2018 for crystal meth (79.6% vs. 67.2%), crack (90.1% vs. 78.4%), heroin (73.5% vs. 59.0%), and fentanyl (67.1% vs. 56.4%).

### Factors associated with crystal meth use

The final analytic sample for assessing factors associated with crystal meth use was limited to 917 respondents after excluding missing data for any of the predictor variables. Survey participants were primarily men (63.0%) and were under 50 years old (74.3%), with a median age of 40 (IQR: 31–50) among participants (Table 2). Most participants were regularly housed (67.3%) and unemployed (79.1%) at the time of survey administration. Furthermore, 19.0% had experienced an opioid overdose and 13.2% had experienced a stimulant overdose in the past six months.

**Table 2:** Characteristics of participants in the 2018 and 2019 Harm Reduction Client Survey (HRCS) stratified by past three-day self-reported crystal meth use (N = 917).

Characteristics^a^	Total (N = 917) n (%)	Crystal meth use (L3D)		P-value^b^
		Yes (N = 623) n (%)	No (N = 294) n (%)	
**Year**				< 0.001
2018	383 (41.8%)	237 (38.0%)	146 (49.7%)	
2019	534 (58.2%)	386 (62.0%)	148 (50.3%)	
**Health authority**				0.065
Fraser Health	306 (33.4%)	216 (34.7%)	90 (30.6%)	
Interior Health	144 (15.7%)	101 (16.2%)	43 (14.6%)	
Island Health	140 (15.3%)	88 (14.1%)	52 (17.7%)	
Northern Health	153 (16.7%)	112 (18.0%)	41 (13.9%)	
Vancouver Coastal Health	174 (19.0%)	106 (17.0%)	68 (23.1%)	
**Urbanicity**				0.993
Small urban/rural communities	293 (32.0%)	199 (31.9%)	94 (32.0%)	
Medium/large urban cities	624 (68.0%)	424 (68.1%)	200 (68.0%)	
**Gender**				0.170
Man	578 (63.0%)	387 (62.1%)	191 (65.0%)	
Woman	322 (35.1%)	221 (35.5%)	101 (34.4%)	
Transgender and gender-expansive^c^	17 (1.9%)	15 (2.4%)	2 (0.7%)	
**Age category**				< 0.001
<50	681 (74.3%)	492 (79.0%)	189 (64.3%)	
≥50	236 (25.7%)	131 (21.0%)	105 (35.7%)	
**Currently regularly housed**				< 0.001
Yes	617 (67.3%)	385 (61.8%)	232 (78.9%)	
No	300 (32.7%)	238 (38.2%)	62 (21.1%)	
**Currently employed**				< 0.001
Yes	192 (20.9%)	109 (17.5%)	83 (28.2%)	
No	725 (79.1%)	514 (82.5%)	211 (71.8%)	
**Opioid use (L3D)**^d^				< 0.001
Yes	536 (58.5%)	422 (67.7%)	114 (38.8%)	
No	381 (41.5%)	201 (32.3%)	180 (61.2%)	
**Opioid agonist treatment use (L3D)**^e^				0.734
Yes	246 (26.8%)	165 (26.5%)	81 (27.6%)	
No	671 (73.2%)	458 (73.5%)	213 (72.4%)	
**Cannabis use (L3D)**				< 0.001
Yes	468 (51.0%)	353 (56.7%)	115 (39.1%)	
No	449 (49.0%)	270 (43.3%)	179 (60.9%)	
**Crack/cocaine use (L3D)**				0.317
Yes	276 (30.1%)	194 (31.1%)	82 (27.9%)	
No	641 (69.9%)	429 (68.9%)	212 (72.1%)	
**Benzodiazepine use (L3D)**				0.177
Yes	103 (11.2%)	76 (12.2%)	27 (9.2%)	
No	814 (88.8%)	547 (87.8%)	267 (90.8%)	
**Alcohol use (L3D)**				0.019
Yes	340 (37.1%)	247 (39.6%)	93 (31.6%)	
No	577 (62.9%)	376 (60.4%)	201 (68.4%)	
**Polysubstance use (L3D)**^f^				< 0.001
Yes	520 (56.7%)	454 (72.9%)	66 (22.4%)	
No	397 (43.3%)	169 (27.1%)	228 (77.6%)	
**Opioid overdose experience (L6M)**				0.004
Yes	174 (19.0%)	134 (21.5%)	40 (13.6%)	
No	743 (81.0%)	489 (78.5%)	254 (86.4%)	
**Stimulant overdose experience (L6M)**				0.014
Yes	121 (13.2%)	94 (15.1%)	27 (9.2%)	
No	796 (86.8%)	529 (84.9%)	267 (90.8%)	

Abbreviations: L3D, Last three days; L6M, Last six months.

^a^ All substances listed refer to self-reported use.

^b^ *P*-values reflect significance of Chi-square test or Fisher’s exact test where appropriate.

^c^ Transgender and gender-expansive includes people that identified as transgender men, transgender women, and gender non-conforming.

^d^ Opioids included heroin, fentanyl, morphine, oxycodone, hydromorphone (Dilaudid).

^e^ Opioid agonist treatment included methadone in 2018 and methadone and/or buprenorphine/naloxone (Suboxone) in 2019.

^f^ Polysubstance use was defined as concurrent use of opioids and stimulants, opioids and benzodiazepines, or stimulants and benzodiazepines.

Participants that reported crystal meth use did not differ significantly in terms of geographic region or urbanicity (Table 2). Those that used crystal meth were relatively younger than those who did not (*P*<0.001), but did not differ in terms of self-reported gender. Additionally, a higher proportion of those that used crystal meth did not have regular housing (38.2% vs. 21.1%; *P*<0.001) and were unemployed (82.5% vs. 71.8%; *P*<0.001).

The majority of participants that reported crystal meth use also reported opioid use in the past three days; opioid use was significantly higher among those that used crystal meth compared to the remaining participants (67.7% vs. 38.8%; *P*<0.001). Lastly, the frequency of reported opioid and stimulant overdose in the past six months was higher among those that used crystal meth (21.5% vs. 13.6%; *P* = 0.004 and 15.1% vs. 9.2%; *P* = 0.014 respectively).

Table 3 presents unadjusted and adjusted odds ratios for factors associated with past three-day self-reported crystal meth use. Health authority, urbanicity, benzodiazepine use, experiencing an opioid overdose, and experiencing a stimulant overdose were not retained in the backward selection process as removing these variables improved model fit. In the multivariate logistic regression model, being 50 years old or greater was associated with decreased odds of crystal meth use (AOR = 0.63; 95% CI: 0.45–0.88) (Table 3). Higher odds of crystal meth use were found among those that did not have regular housing (AOR = 2.08; 95% CI: 1.46–2.97) and were unemployed (AOR = 1.75; 95% CI: 1.21–2.52). In addition, opioid use was also associated with higher odds of crystal meth use (AOR = 3.13; 95% CI: 2.29–4.26), as was cannabis use (AOR = 2.10; 95% CI: 1.53–2.88), and to a lesser degree, alcohol use (AOR = 1.41; 95% CI: 1.02–2.94). Final model estimates were of the same magnitude and direction when verified using all available data (N = 1,107), which included participants that had missing responses or indicated “prefer not to say” (S1 Table).

**Table 3:** Unadjusted and adjusted odds ratios and 95% confidence intervals for factors associated with past three-day self-reported crystal meth use (N = 900).

Characteristics^a^	OR (95% CI)	AOR (95% CI)	*P*-value
**Study year**			
2018	1.00	1.00	-
2019	1.61 (1.21–2.13)	1.66 (1.22–2.26)	0.001
**Age**			
<50	1.00	1.00	-
≥50	0.48 (0.35–0.66)	0.63 (0.45–0.88)	0.007
**Gender**			
Man	1.00	1.00	-
Woman	1.08 (0.81–1.45)	1.05 (0.76–1.46)	0.771
**Currently Regularly Housed**			
Yes	1.00	1.00	-
No	2.35 (1.70–3.26)	2.08 (1.46–2.97)	< 0.001
**Currently Employed**			
Yes	1.00	1.00	-
No	1.96 (1.41–2.73)	1.75 (1.21–2.52)	0.003
**Opioid Use (L3D)**^b^			
Yes	3.40 (2.54–4.55)	3.13 (2.29–4.26)	< 0.001
No	1.00	1.00	
**Cannabis Use (L3D)**			
Yes	1.97 (1.48–2.61)	2.10 (1.53–2.88)	< 0.001
No	1.00	1.00	
**Alcohol Use (L3D)**			
Yes	1.39 (1.03–1.86)	1.41 (1.02–1.94)	0.040
No	1.00	1.00	

Abbreviations: L3D, last three days; OR, odds ratio, AOR, adjusted odds ratio; CI, confidence interval.

^a^ All substances listed refer to self-reported use.

^b^ Opioids included heroin, fentanyl, morphine, oxycodone, and hydromorphone (Dilaudid).

### Validity of self-reported crystal meth use

A total of 316 participants provided a urine sample for urine toxicology screening in 2018, and 601 in 2019. Of these, 40 samples could not be linked with corresponding survey data (3 in 2018 and 37 in 2019) due to missing survey records or exclusion due to participant age less than 19, leaving a final sample of 877 participants for assessing validity of self-reported crystal meth use. Among participants, 77% (n = 677) had methamphetamine detected in their urine (true prevalence) and 69.0% (n = 609) reported using crystal meth in the past three days (apparent prevalence).

Measures of validity for past three-day self-reported crystal meth use compared with urine toxicology screening are provided in Table 4. Overall, sensitivity of self-reported crystal meth use was 86% [95% CI: 83%–88%], specificity was 86% [95% CI: 81%–91%], which translated to a positive predictive value of 96% [95% CI: 94%–97%], and a negative predictive value of 65% [95% CI: 59%–70%]. Positive likelihood ratio was 6.37 (95% CI 4.48–9.06), and negative likelihood ratio was 0.16 (95% CI 0.13–0.20).

**Table 4:** Measures of validity and 95% confidence intervals for self-reported past three-day methamphetamine use compared to urine toxicology screening (N = 877).

Test	Test value	95% CI
Sensitivity	86%	(83%– 88%)
Specificity	86%	(81%– 91%)
Positive predictive value	96%	(94%– 97%)
Negative predictive value	65%	(59%– 70%)
Positive likelihood ratio	6.37	(4.48–9.06)
Negative likelihood ratio	0.16	(0.13–0.20)

Abbreviations: CI, confidence interval.

## Discussion

In this cross-sectional study among people who accessed harm reduction services in BC in 2018 and 2019, crystal meth was the most commonly reported drug used in the previous three days, more than opioids, cannabis, or alcohol. Its use was strongly associated with opioid use, cannabis use, and alcohol use, and use of other substances was frequently reported among those that used crystal meth. The frequency of use was significantly higher in people who were under 50 years of age, unhoused, and unemployed.

The prevalence of crystal meth use in 2018 and 2019 was significantly greater than that reported in the previous iteration of the harm reduction client survey. In 2015, aside from cannabis, crystal meth use was on par with heroin for the most commonly used drugs, with 47% percent of participants reporting use [32]. Thus, crystal meth use has increased significantly and surpassed illegal opioids, including heroin and fentanyl, to be the most prevalent drug used in 2019. These findings are corroborated among street-involved adults who use substances in Victoria, BC, where crystal meth use steadily increased between 2010 and 2015 [33]. Similarly, findings from two prospective cohorts of people who inject drugs in Vancouver, BC, demonstrated a 17% increase in crystal meth use between 2006 and 2017 in both men and women [20]. This increase has also been noted through a concomitant rise in proxy indicators of crystal meth use in North America [7, 8], including an increase in detection of methamphetamine among drug toxicity deaths in BC [34].

While the precise reasons for the increase is not clear, studies have pointed to several factors that may have influenced the increased use of crystal meth including greater availability, lower cost, and increased product purity [4]. Fentanyl and methamphetamine are consistently amongst the top three identified drugs by Health Canada’s Drug Analysis Service seized by law enforcement in BC [35]. Some jurisdictions have also reported greater availability of crystal meth in times of low or restricted heroin or opioid availability [4, 36, 37]. Additionally, others have posited that crystal meth use may be related to a necessity to stay alert and awake when experiencing periods of homelessness [3, 20], particularly among youth experiencing homelessness [17]. Individuals facing homelessness may need to stay alert to protect belongings and prevent harassment [38]. This function of crystal meth use aligns with findings of the current study where being unhoused was associated with higher odds of crystal meth use.

Other reasons for the increased prevalence of crystal meth use may be related to increased use among people who use opioids and opioid agonist treatment, such as methadone. In the current study, the prevalence of crystal meth use was much higher among those who used opioids in the last three days compared to those who did not use opioids, and opioid use was significantly associated with crystal meth use. Moreover, two-thirds of those that had used opioid agonist treatment in the past three days also reported use of crystal meth. In many cohorts, polysubstance use with both methamphetamine or similar stimulants and heroin or similar opioids is the dominant pattern of use [3, 4, 39, 40]. Crystal meth and opioid polysubstance use has been discussed as a means of achieving a balance between the sedating effects of opioids and excess stimulant excitability, or as a method of achieving a combined, ‘synergistic high’ or euphoria [3, 4, 37, 41, 42]. Use of crystal meth to combat sedating effects of opioids and opioid agonist treatment may also be a pragmatic necessity for individuals that need to resume income-generating activities [4, 42]. While unemployment was significantly associated with higher odds of crystal meth use in the current study, more than half of the participants that were employed also used crystal meth.

Understanding patterns of crystal meth use is vital considering harms associated with its use and limited harm reduction services and interventions available for those that use crystal meth. Additionally, the combination of crystal meth and opioid use may present compounded harms, including increased frequency of injection and unsafe injection practices, increased risk of non-fatal overdose, and relapse to opioid use for those on opioid agonist treatment [3, 15, 16, 43, 44]. In the current study, prevalence of both opioid and stimulant overdose in the past six months was higher among those that used crystal meth when compared to those that did not. Increased detection of crystal meth and metabolites among drug toxicity deaths in BC [34] highlights a need to better understand the interaction of crystal meth and opioids, and how they may act to affect risk of overdose. Given the high prevalence of co-use of crystal meth and opioids, further development and evaluation of therapeutic options for stimulant use disorder and opioid use disorder must occur in the context of polysubstance use rather than in isolation.

While focus of research and harm reduction messaging and programming is often attentive to mitigating harms associated with injecting substances, the current study found that smoking was the most commonly used route of administration for both stimulants and opioids. The focus on injection practices may have inadvertently created a gap in knowledge and response to harms that may arise from smoking substances, including how it affects the risk of overdose, patterns of drug use, and a lack of services and education tailored to individuals who prefer to smoke substances. Previous findings from the Harm Reduction Client Survey, for instance, demonstrated that individuals that preferred smoking were less likely to carry a naloxone kit for opioid overdose response [22]. Understanding the risk associated with different patterns of use is crucial in recognizing where efforts to prevent morbidity and mortality can be directed. As safer injection supplies are often more readily available than safer smoking supplies [45], harm reduction services must also adapt to changing patterns of substance use and meet the need for distribution of safer smoking supplies, including crystal meth pipes.

Comparison of survey data to urine toxicology screening demonstrated relatively high sensitivity of self-report and three-day recall of crystal meth use. While previous studies have reported variable sensitivity of self-reported crystal meth use [46–48], validity measures in the current study were comparable to those reported among adults enrolled in a randomized controlled pharmacotherapy trial for methamphetamine dependence in the US [48]. The high sensitivity of self-reported crystal meth use may be due to participants feeling more comfortable disclosing substance use anonymously, with no potential for linkage with identifiable information. Additionally, participants may be less likely to experience stigma associated with substance use at harm reduction supply distribution sites [49], potentially limiting social desirability bias in their responses. The validity measures reported here should thus be interpreted within the context and setting of the study, and may not be generalizable to larger population-based surveys or studies in clinical treatment settings, where abstinence from substance use may be expected.

Our work adds to the existing literature on crystal meth epidemiology in three main ways. First, it provides a large, contemporary, and more generalizable estimate of crystal meth use in BC, building on previous work that has focused on youth and urban populations. Second, it provides evidence on use patterns among all harm reduction service clients, not limited to those who use opioids. Third, it reports on the accuracy of self-reported crystal meth use which can be helpful to clinicians and patients when providing health services. As with all survey methods, the data are limited in quality by self-reporting and as they are cross-sectional, we cannot assess causal associations. Limitations around ability to recall were mitigated by asking about recent (three-day) substance use and substance use patterns, and validity of self-reported crystal meth use for wider recall windows may differ. Furthermore, we did not have access to additional information on family history of substance use and duration of substance use, which may be important correlates to consider. Additionally, interpretations of the current study are limited by the nature of the sample which represents people who use substances that are accessing harm reduction services, and may not be generalizable to the entire population of people who use substances in BC and elsewhere.

## Conclusions

Crystal meth is the most commonly used drug among harm reduction clients in BC and its use has increased over time. Its use is highly prevalent among those facing homelessness and often closely intertwined with opioid use, increasing risk of overdose and other sequelae associated with polysubstance use. Investment in harm reduction services and health services for the prevention and management of harms that may arise from crystal meth use is necessary, in addition to prospective epidemiological research to better understand the patterns of use, dependence, and health and social outcomes.

## Supporting information

S1 Appendix 2018 British Columbia Harm Reduction Client Survey. (PDF)

S2 Appendix 2019 British Columbia Harm Reduction Client Survey. (PDF)

S1 Table Unadjusted and adjusted odds ratios and 95% confidence intervals for factors associated with past three-day self-reported crystal meth use (N = 1,107). (DOCX)
