# Supplementary material for: Crystal methamphetamine use in British Columbia, Canada: A cross-sectional study of people who access harm reduction services
Source: PLoS One. 2021 May 26;16(5):e0252090. doi: 10.1371/journal.pone.0252090 (PMC8153500; doi:10.1371/journal.pone.0252090)
Supplement: S1 Appendix — (PDF) [file pone.0252090.s001.pdf]

2018 Harm Reduction Client Survey

Harm Reduction Services and Strategies is conducting a survey to help improve harm reduction services across BC. No personal identifying information will be collected and your responses will be kept confidential. Your participation is voluntary and you are free to only answer the questions you are comfortable with. The survey will take roughly 20 minutes of your time. Please note that you can only complete the survey once.

To participate in this survey you must have used an illegal drug other than cannabis within the last 6 months.

1. What is your current GENDER identity? (Select one)
❑ Female ❑ Male ❑ Trans man ❑ Trans woman ❑Gender non-conforming ❑ Other, specify: ______________ ❑ Prefer not to say

2. How old are you? ____________ (years)
❑ Prefer not to say

3. Which best describes your sexual orientation? (Select one)
❑ Gay ❑ Lesbian ❑ Straight ❑ Bisexual ❑ Queer ❑ Other, specify: ______________ ❑ Prefer not to say

4. Do you identify yourself as First Nations? (Select one)
❑ Yes *(continue)* ❑ No *(skip to #6)* ❑ Prefer not to say *(skip to #6)*

↳ **5a. If you identify as a First Nations person, do you currently live on a reserve?** (Select one)
❑ Yes ❑ No ❑ Prefer not to say

↳ **5b. If you identify as a First Nations person, are you:** (Select one)
❑ Status ❑ Non-status ❑ Prefer not to say

6. Do you identify yourself as Métis or Inuit? (Select all that apply)
❑ Yes, Métis ❑ Yes, Inuit ❑ No ❑ Prefer not to say

7. Do you currently live in: ___________________ (Harm reduction site location)? (Select one)
❑ Yes ❑ No, I live in (specify city): ______________________ ❑ Prefer not to say

8. How long have you lived at your current address? (Select one)
❑ More than 1 year ❑ 7-12 months ❑ 1-6 months ❑ Less than 1 month
❑ I have no regular place to stay (homeless, shelter, couch surf, No Fixed Address) (Skip to #10) ❑ Prefer not to say

↳ **9a. Do you live alone?** (Select one)
❑ Yes ❑No, I live with roommates ❑ No, I live with relatives ❑Other, Specify ___________❑ Prefer not to say

10. Are you currently employed? (Select all that apply)
❑ Yes, paid volunteer ❑ Yes, part -time ❑ Yes, full-time ❑No ❑ Prefer not to say

11. How did you get here today? (Select all that apply)
❑ Walked ❑ Biked ❑ Drove Myself ❑ Someone drove me ❑ Taxi
❑ Bus/ Skytrain/ Transit ❑ Mobile Site / Outreach came to me ❑ Prefer not to say

12. How long, in total, did it take you to get here today? (Select one)
❑ 0 minutes – Outreach came to me ❑ 1 - 10 minutes ❑ 11 - 30 minutes
❑ 31 - 60 minutes ❑ Over 1 hour ❑ Prefer not to say

13. Do you have a cellphone? (Select one)
❑ Yes (continue) ❑ No *(Skip # 15)* ❑ Prefer not to say

↳ **14a. Does your cellphone have the capability to make a call right now?** (Select one)
❑ Yes, I have pre-paid minutes ❑ Yes, I have a monthly plan ❑ No, I don't have minutes or a plan
❑ Prefer not to say

↳ **14b. Do you have access to the internet on your phone? :** (Select one)
❑ Yes, I have a data plan ❑ Yes, but I don't have any data ❑ No *(Skip to # 15)* ❑ Prefer not to say

↳ **14c. Would you use any of the following apps on your phone?** (Select all the apply)
❑ App to report tainted drug supply ❑ App to receive overdose and drug supply alerts
❑ App to alert bystanders with naloxone ❑ App that uses your smartphone camera to monitor your breathing while using drugs
❑ Other: ____________ ❑ I wouldn't use an app ❑ Prefer not to say

15. Are you here *TODAY* to… (Select all that apply)
❑ Pick up supplies for myself ❑ Pick up supplies for someone else ❑ Access health or other services
❑ Other, specify: ___________________________ ❑ Pick up a naloxone kit ❑ Prefer not to say

16. In the last *month*, have you picked up supplies (e.g. needles) from any site/outreach, either for yourself or another person?(Select one)
❑ Yes ❑ No *(skip to #18)* ❑ Prefer not to say *(skip to #18)*

↳ **17a. In the last month, how often would you say you picked up supplies from any site/outreach?** (Select one)
❑ Every day ❑ A few times a week ❑ A few times a month ❑ Less than once a month ❑ Prefer not to say

↳ **17b. In the last month, did any of the following make it difficult for you to pick up supplies from any site/outreach?** (Select all that apply)
❑ Site not open ❑ Site too far away ❑ Staff had negative attitudes ❑ Concerned about confidentiality
❑ Site didn't have the supplies I needed, specify:_________________
❑ Other, specify: ____ ❑ Prefer not to say

↳ **17c. Which of these would you be comfortable accessing through a community pharmacy?** (Select all that apply)
❑Smoking supplies (e.g. for crack, meth, heroin) ❑ Injection supplies ❑ Naloxone kit ❑ None ❑Prefer not to say

18. In the last 6 months, how often would have you safely disposed of supplies at any site/outreach/drop box? (Select one)
❑ A few times a week ❑A few times a month ❑Less than once a month ❑ Never ❑Prefer not to say

↳ **19a. In the 6 month, did you encounter barriers to disposing your supplies in your community** (Select one)
❑ Yes ❑ No *(skip to #20)* ❑ Prefer not to say

↳ **19b. What were the barriers to disposing your supplies?**
❑ Worried about being stigmatized ❑ Not enough disposal locations nearby ❑Disposal sites hours were too short
❑ Other, Specify _____________ ❑ Prefer not to say

20. In the last month, have you injected any type of drug? (Select one)
❑ Yes ❑ No *(skip to #24)* ❑ Prefer not to say *(skip to #24)*

↳ **21a. In the last month, did you have any trouble getting unused needles?** (Select one)
❑ Yes ❑ No ❑ Prefer not to say

↳ **21b. In the last month, have you ever fixed with a rig that had been used by someone else?** (Select one)
❑ Yes ❑ No ❑ Prefer not to say

22. In the last month, have you injected drugs at an overdose prevention site (OPS)? (Select one)

An OPS is a place (fixed or mobile) where drug consumption is supervised by staff or volunteers to reduce overdose related deaths.
❑ Yes ❑ No *(Skip to # 23c)*

↳ **23a. In the last month, what type of overdose prevention site (OPS) have you used? (Select all the apply)**
❑ Shelter or housing ❑ Community Health Centre / Health Clinic ❑ Stand-alone facility (like Insite)
❑ Mobile Site ❑ Community Organization (like VANDU) ❑ Other, specify: ____________________

↳ **23b. How often are you using an overdose prevention site (OPS)? (Select one)**
❑ Every day *(Skip to #23e)* ❑ A few times a week *(Skip to #23e)* ❑ A few times a month *(Skip to #23e)*
❑ Less than once a month *(Skip to #23e)* ❑ Prefer not to say *(Skip to #23e)*

↳ **23c. Why haven't you used an of overdose prevention site (OPS)? (Select one)**
❑ Service not available nearby *(continue # 23d)* ❑ Worried about being stigmatized at OPS ❑ Staff had negative attitudes
❑ Concerned about confidentiality ❑ Other, Specify: ______________________ ❑ Prefer not to say

↳ **23d. If it were made available to you, which setting would you use for an overdose prevention site (OPS)?** (Select all that apply)
❑ Shelter or housing ❑ Community Health Centre / Health Clinic ❑ Stand-alone facility (like Insite)
❑ Mobile Site ❑ Other, specify: ____________________ ❑ I wouldn't use a OPS ❑ Prefer not to say

↳ **23e. Which drug consumption methods should be allowed at an overdose prevention site (OPS)?** (Select all that apply)
❑ Injection ❑ Inhalation/smoking ❑ Snorting ❑ Other, Specify: _________________ ❑ Prefer not to say

24. In the last year, have you been involved with any grassroots drug user groups (NOT support groups)? (Select all that apply)
❑ CAPUD – Canadian Association of People who Use Drugs ❑ BC/Yukon Association of Drug War Survivors
❑ BCAPOM – BC Association of People on Methadone ❑ REDUN - Rural Empowered Drug User Network
❑ SOLID – Society of Living Illicit Drug Users ❑ VANDU - Vancouver Area Network of Drug Users
❑ WAHRS - Western Aboriginal Harm Reduction Society ❑ Other user group in your community: ________________________
❑ Other user group in Canada:_________________ ❑ Prefer not to say

25. In the last 6 months, have you had difficulty accessing Opioid Substitution Treatment (OST)/Opioid agonist therapy (OAT)?(Select one)
OST/OAT are drug therapies that counters opioid withdrawal symptoms and acts as a substitute for the opioids you were previously taking
❑ No, I did not have difficulty *(skip to #27)* ❑ No, I did not try to access OST/OAT*(skip to #27)*
❑ Yes, I had difficulty *(continue)* ❑ Prefer not to say *(skip to #27)*

↳ **26. If yes, what was the difficulty?** (Select all that apply)
❑ Could not find a prescribing physician ❑ There were no pharmacies nearby
❑ Could not get prescription because of positive urine test ❑ Clinic fees were too high
❑ Worried about being stigmatized at clinic ❑ Wasn't offered preferred OST/OAT
❑ Other, specify: _________________________ ❑ Prefer not to say

27. Would you be interested in the supervised OAT hydromorphone injectable at your community pharmacy? (Select all that apply)
This would mean 2-3x daily injection of hydromorphone in a booth at the pharmacy under prescription from a doctor
❑ Yes ❑ No ❑ prefer not to say

28. In the last 6 months, were you taking any of the following Opioid Substitution Treatment (OST)? (Check all that apply)
❑ Methadose ❑ Suboxone (buprenorphine/naloxone) ❑ Kadian (Slow-Release Oral Morphine)
❑ Injected liquid Dilaudid (hydromorphone) ❑ Dilaudid (hydromorphone) in pill form ❑ Prefer not to say

↳ **26a. In the last 6 months, did you discontinue Opioid Substitution Treatment (OST)?** (Select one)
❑ Yes ❑ No *(skip to # 29)* ❑ Prefer not to say *(skip to # 29)*

↳ **26b. Why did you discontinue Opioid Substitution Treatment (OST)?** *(Open ended – Write response)*

**29. Have you used any of these in the last 3 days?** (circle No or Yes for each) If Yes, continue table→			**Did you use it the last 7 days?**		**How did you use it?** (Circle all that apply)			**Do you usually have a prescription for it?**	
**Cannabis / Hash**	No	Yes →	No	Yes	Smoke		Other	No	Yes
**Methadone / Methadose**	No	Yes →	No	Yes	Smoke	Inject	Other	No	Yes
**Morphine**	No	Yes →	No	Yes	Smoke	Inject	Other	No	Yes
**Dilaudid**	No	Yes →	No	Yes	Smoke	Inject	Other	No	Yes
**Oxycodone**	No	Yes →	No	Yes	Smoke	Inject	Other	No	Yes
**Fentanyl**	No	Yes →	No	Yes	Smoke	Inject	Other	No	Yes
**Xanax**	No	Yes →	No	Yes	Smoke	Inject		No	Yes
**Other Benzos (Ativan/ Valium)**	No	Yes →	No	Yes	Smoke	Inject	Other	No	Yes
**Stimulant (Ritalin /Adderall)**	No	Yes →	No	Yes	Smoke	Inject	Other	No	Yes
**Crystal Meth**	No	Yes →	No	Yes	Smoke	Inject	Other		
**Cocaine (powder)**	No	Yes →	No	Yes	Smoke	Inject	Other		
**Crack**	No	Yes →	No	Yes	Smoke	Inject	Other		
**Heroin**	No	Yes →	No	Yes	Smoke	Inject	Other		
**Tobacco (cigarettes)**	No	Yes →	No	Yes	Smoke	Chew	Other		
**Alcohol**	No	Yes →	No	Yes					
**Other 1:**	No	Yes →	No	Yes	Smoke	Inject	Other	No	Yes
**Other 2:**	No	Yes →	No	Yes	Smoke	Inject	Other	No	Yes

30. In the last 7 days, what percentage of the time did you intentionally use more than 1 drug at a time? (Select one)
❑ 0% of the time ❑ 1- 25% of the time ❑ 26- 50% of the time ❑ 51-75% of the time
❑ 100%of the time ❑ Prefer not to say

31. What is your preferred method of using drugs? (Select one)
❑ Injection ❑ Inhalation/smoking ❑ Snorting ❑ Other, Specify: _________________ ❑ Prefer not to say

32. In the last 7 days, what percentage of the time did you use drugs alone? (Select one)
❑ 0% of the time *(skip to # 34)* ❑ 1- 25% of the time ❑ 26-50% of the time
❑ 51-75% of the time ❑ 100% of the time ❑ Prefer not to say *(skip to #34)*

↳ **33. What are some of the reasons you use drugs alone?** (select all the apply)

❑ It's safer to be alone ❑ It's more convenient and comfortable to use at home ❑ I don't want others to know that I'm using drugs
❑ Other ______________________________ ❑ Prefer not to say

34. In the last 6 months, have you intentionally used Fentanyl? (Select all that apply)
❑ Yes, patches ❑ Yes, pills ❑ Yes, powder ❑ No *(skip to #36)* ❑ Prefer not to say *(skip to #36)*

↳ **35. How much did Fentanyl cost the MOST RECENT time you purchased it?**
$ ________ per patch ❑ Don't know or don't remember
$ ________ per tablet or capsule ❑ Prefer not to say
$ ________ for powder per _______ (quantity)

36. In the last 6 months, have you un-intentionally used Fentanyl? (select one)
❑ Yes ❑ No *(Skip to #38)* ❑ Don't know ❑ Prefer not to say *(Skip to #38)*

↳ **37. In the last month, how often have you used a substance that you believe also contained Fentanyl?** (Select One)
❑ 0% of the time ❑ less than 30% of the time ❑ 50% of the time ❑ 80% of the time ❑ 100% of the time ❑ Prefer not to say

38. If you had your drugs tested and they tested positive for fentanyl would you change the amount you were going to use? (Select One)
❑ Yes, would use less ❑ Yes, would use more ❑ No, nothing would change ❑ Prefer not to say

39. In the last month, have you used a glass (meth or crack) pipe from a harm reduction site to smoke any drug? (Select one)
❑ Yes ❑ No *(skip to #41)* ❑ Prefer not to say *(skip to #41)*

↳ **40. In the last month, what did you do when you couldn't get new / unused pipes to smoke any drug?** (Select all that apply)
❑ Injected instead ❑ Waited until I could find a new pipe ❑ Shared, bought, or borrowed a used pipe
❑ Smoked without a pipe using (specify):______________________ ❑ Snorted/swallowed instead
❑ I did not have a problem getting pipes ❑ Prefer not to say

41. In the last 6 months, have YOU overdosed (aka overamped) by accident from using any stimulant (e.g Crack, Crystal Meth)? (Select one)
❑ Yes ❑ No *(skip to #43)* ❑ Don't know *(skip to #43)* ❑ Prefer not to say *(skip to #43)*

↳ **42a. During your most recent stimulant overdose what were your symptoms?** (select all that apply)
❑ Nausea/ Vomiting ❑ Falling asleep/passing out (but still breathing) ❑ Chest pain ❑ High Temperature/sweating profusely
❑ Racing pulse ❑ Irregular Breathing or shortness of breath ❑ Extreme anxiety ❑ Feeling paralyzed but awake
❑ Seizure ❑ Jerking or rigid limb ❑ Stroke ❑ Don't know ❑ Prefer not to say

↳ **42b. In the last 6 months, when you had the most recent stimulant overdose were you given Naloxone/Narcan?** (Select one)
❑ Yes ❑ No *(skip to #43)* ❑ Don't know *(skip to #43)* ❑ Prefer not to say *(skip to #43)*

↳ **42c. When you were given Naloxone/Narcan for a stimulant overdose most recently, was it given to you by:** (Select one)
❑ Paramedic or nurse or health worker ❑ OPS Staff ❑ Friend / family member ❑ Housing worker
❑ Stranger who happened to be there ❑ Don`t know ❑ Other, specify____________ ❑ Prefer not to say

43. In the last 6 months, have YOU overdosed by accident from using any opioids, such as heroin or morphine? (Select one)
❑ Yes ❑ No *(skip to #45)* ❑ Don't know *(skip to #45)* ❑ Prefer not to say *(skip to #45)*

↳ **44a. In the last 6 months, when you had the most recent opioid overdose were you given Naloxone/Narcan?** (Select one)
❑ Yes ❑ No *(skip to #45)* ❑ Don't know *(skip to #45)* ❑ Prefer not to say *(skip to #45)*

↳ **44b. When you were given Naloxone/Narcan most recently, was it given to you by:** (Select one)
❑ Paramedic or nurse or health worker ❑ OPS Staff ❑ Friend / family member ❑ Housing worker
❑ Stranger who happened to be there ❑ Don`t know ❑ Other, specify____________ ❑ Prefer not to say

45. In the last 6 months, have you SEEN an accidental overdose in someone using any opioids? (Select one)
❑ Yes ❑ No *(skip to #47)* ❑ Don't know *(skip to #47)* ❑ Prefer not to say *(skip to #47)*

↳ **46a. In the last 6 months, did you give Naloxone/Narcan to someone experiencing an overdose?** (Select one)
❑ Yes *(skip to #47)* ❑ No ❑ Don't know *(skip to #47)* ❑ Prefer not to say *(skip to #47)*

↳ **46b. Why did you not give Naloxone/Narcan to the person experiencing an overdose?** (Select one)
❑ Don't know how to use Naloxone/Narcan ❑ Naloxone/Narcan was not available
❑ Other, specify:______________________________ ❑ Prefer not to say

47. Do you have a Naloxone/Narcan kit? (Select one)
❑ Yes ❑ No, I do not have a kit but I want one *(Continue)* ❑ No, I do not have a kit and I do not want one ❑ Prefer not to say

↳ **48a. What were the barriers accessing a Naloxone/Narcan kit?**
❑ Worried about being stigmatized ❑ I don't know where to access a kit ❑ The site to access a kit is too far away
❑ Other, Specify ______________________________ ❑ Prefer not to say

49. Have you ever been tested for hepatitis C virus (HCV) infection? (select one)
❑ Yes ❑ No *(Skip to #51)* ❑ Don't know ❑ Prefer not to say *(Skip to #51)*

↳ **50a. If yes, when did you last get tested for HCV?** (Select One)
❑ Within the 12 months ❑ 2-5 years ago ❑ Over 5 years ago ❑ Prefer not to say

↳ **50b. If yes, did you get further follow-up and care for that.** (Select one)
❑ Yes ❑ No ❑ Prefer not to say

51. *This next question is about participating in a future study.*
Would you be willing to participate in a finger prick test for Hepatitis C (HCV)? These results would not be shared with you but would inform the prevalence of undiagnosed HCV in your region. (Select one)
❑ Yes ❑ No ❑ Prefer not to say

Thank you for taking the survey!

Place ID # Sticker Here
