# Supplementary material for: Crystal methamphetamine use in British Columbia, Canada: A cross-sectional study of people who access harm reduction services
Source: PLoS One. 2021 May 26;16(5):e0252090. doi: 10.1371/journal.pone.0252090 (PMC8153500; doi:10.1371/journal.pone.0252090)
Supplement: S2 Appendix — (PDF) [file pone.0252090.s002.pdf]

Place ID #
Sticker Here

2019 Harm Reduction Client Survey

This survey is being conducted to help improve harm reduction services. No personal identifying information will be collected and your responses will be kept confidential. Your participation is voluntary and you are free to only answer the questions you are comfortable with. The survey will take roughly 20 minutes of your time. Please note that you can only complete the survey once.

To participate in this survey you must have used an illegal drug other than cannabis within the last 6 months.

1. What is your current GENDER identity? (Select one)
❑ Woman ❑ Man ❑ Trans man ❑ Trans woman ❑ Gender non-conforming
❑ Other, specify: _______________ ❑ Prefer not to say

2. How old are you? ______________ (years) ❑ Prefer not to say

3. Do you identify yourself as any of the following? (Select one)
❑ First Nations ❑ Métis ❑ Inuit ❑ No ❑ Prefer not to say

4. Do you live: (Select one)
❑ In a private residence, alone ❑ In a private residence, with someone else
❑ Other residence (hotels, motels, rooming houses, single room occupancy (SRO), shelters, social/supportive housing etc.)
❑ I have no regular place to stay (homeless, couch surf, No Fixed Address)
❑ Other, specify _____________ ❑ Prefer not to say

5. How long has this been your living situation? (Select one)
❑ More than 1 year ❑ 7-12 months ❑ 1-6 months ❑ Less than 1 month ❑ Prefer not to say

6. Are you currently employed? (Select all that apply)
❑ Yes, paid volunteer ❑ Yes, part -time ❑ Yes, full-time ❑ No ❑ Prefer not to say

7. Do you have a cellphone? (Select one)
❑ Yes ❑ No ❑ Prefer not to say

8. How did you get here today? (Select one)
❑ Walked ❑ Biked ❑ Drove Myself ❑ Bus/ Skytrain/ Transit ❑ Taxi
❑ Someone drove me ❑ Mobile Site / Outreach came to me ❑ Prefer not to say

9. How long, in total, did it take you to get here today? (Select one)
❑ Outreach came to me ❑ 1 - 10 minutes ❑ 11 - 30 minutes
❑ 31 - 60 minutes ❑ Over 1 hour ❑ Prefer not to say

**10. Did you use any of these in the LAST 3 DAYS?**			**IF YES ➔ (Circle all that apply)**						
			**How did you use it?**					**Did you have a prescription for it?**	
**Methadone (Methadose/Metadol)**	No	Yes	Smoke	Snort	Inject	Swallow	Other	No	Yes
**Buprenorphine/Naloxone (Suboxone)**	No	Yes	Smoke	Snort	Inject	Swallow	Other	No	Yes
**Hydromorphone (Dilaudid)**	No	Yes	Smoke	Snort	Inject	Swallow	Other	No	Yes
**Oxycodone**	No	Yes	Smoke	Snort	Inject	Swallow	Other	No	Yes
**Morphine**	No	Yes	Smoke	Snort	Inject	Swallow	Other	No	Yes
**Heroin**	No	Yes	Smoke	Snort	Inject	Swallow	Other	No	Yes
**Fentanyl**	No	Yes	Smoke	Snort	Inject	Swallow	Other	No	Yes
**Xanax**	No	Yes	Smoke	Snort	Inject	Swallow	Other	No	Yes
**Other Benzos (Ativan/Valium)**	No	Yes	Smoke	Snort	Inject	Swallow	Other	No	Yes
**Stimulant (Ritalin/Adderall)**	No	Yes	Smoke	Snort	Inject	Swallow	Other	No	Yes
**Crystal Meth/Methamphetamine**	No	Yes	Smoke	Snort	Inject	Swallow	Other		
**Cocaine (powder)**	No	Yes	Smoke	Snort	Inject	Swallow	Other		
**Crack**	No	Yes	Smoke	Snort	Inject	Swallow	Other		
**MDMA**	No	Yes	Smoke	Snort	Inject	Swallow	Other		
**Cannabis/Hash**	No	Yes	Smoke	Snort	Inject	Swallow	Other	No	Yes
**Tobacco (cigarettes)**	No	Yes	Smoke	Snort	Chew	Swallow	Other		
**Alcohol**	No	Yes				Swallow			
**Other 1:**	No	Yes	Smoke	Snort	Inject	Swallow	Other	No	Yes
**Other 2:**	No	Yes	Smoke	Snort	Inject	Swallow	Other	No	Yes

11. In the last 3 days, did you use both uppers (e.g. crystal meth) and downers (e.g. heroin) one after the other or together? (Select all that apply)
❑ No ❑ Yes, downers then uppers. If so, specify why: ______________________________
❑ Yes, uppers then downers. If so, specify why: ______________________________
❑ Yes, I mix uppers and downers together. If so, specify why: ______________________________
❑ Other, specify:______________________________ ❑ Prefer not to say

12. In the *__past month__*, how often did you use drugs by any mode (excluding cannabis, alcohol, or tobacco)?
❑ Every day ❑ A few times a week ❑ A few times a month ❑ Prefer not to say

13. If you use down, what would you prefer to use? (Select one)
❑ Heroin ❑ Fentanyl ❑ Morphine ❑ Oxycodone
❑ Hydromorphone (Dilaudid) ❑ Methadone/Methadose ❑ Buprenorphine/naloxone (Suboxone)
❑ I don't use down ❑ Prefer not to say

14. If you use uppers/stimulants, what would you prefer to use? (Select one)
❑ Crystal Meth/Methamphetamine ❑ Cocaine (powder) ❑ Crack ❑ MDMA
❑ Stimulants (Ritalin/Adderall) ❑ I don't use stimulants ❑ Prefer not to say

15. If your drugs tested positive for fentanyl (before you use), would you change the amount you use? (Select One)
❑ Yes, would use less ❑ Yes, would use more ❑ No, nothing would change ❑ Prefer not to say

16. What is your preferred method of using drugs? (Select one)
❑ Smoking/inhalation ❑ Snorting ❑ Injecting ❑ Swallowing
❑ Other, Specify: ______________________ ❑ Prefer not to say

17. How often do you use drugs alone? (Select one)
❑ Never ❑ Occasionally ❑ Often ❑ Always ❑Prefer not to say

18. What are some of the reasons YOU use drugs alone? (Select all that apply)
❑ It's safer to be alone ❑ It's more convenient and comfortable to use at home
❑ I don't want to share ❑ I don't want others to know that I'm using drugs
❑ I don't have anyone else to use with ❑ I never use alone
❑ Other, Specify: ____ ❑ Prefer not to say

19. In the __last 6 months__, have YOU overdosed (overamped) by accident from using a stimulant (eg. Crystal meth, crack)? (Select one)
❑ Yes ❑ No ❑ Don't know ❑ Prefer not to say

20. In the __last 6 months__, have YOU overdosed by accident from using any opioids (eg. fentanyl, heroin)? (Select one)
❑ Yes ❑ No *(skip to #22)* ❑ Don't know *(skip to #22)* ❑ Prefer not to say *(skip to #22)*

21a. In the __last 6 months__, how many times did you overdose by accident from using opioids? (Select one)
❑ Once ❑ 2 times ❑ 3 times ❑ 4 times ❑ 5 or more times ❑ Prefer not to say

21b. In the __last 6 months__, when you had the most recent opioid overdose were you given Naloxone/Narcan? (Select one)
❑ Yes ❑ No *(skip to #22)* ❑ Don't know *(skip to #22)* ❑ Prefer not to say *(skip to #22)*

21c. When you were given Naloxone/Narcan most recently, was it given to you by: (Select all that apply)
❑ Paramedic or emergency responder ❑ OPS/ SCS Staff ❑ Friend / family member
❑ Stranger who happened to be there ❑ Housing worker ❑ Nurse/health worker in hospital
❑ Other, specify______________ ❑ Don`t know ❑ Prefer not to say

22. In the __last 6 months__, have you SEEN an accidental overdose in someone using any opioids? (Select one)
❑ Yes ❑ No *(skip to #24)* ❑ Don't know *(skip to #24)* ❑ Prefer not to say *(skip to #24)*

23a. Did you give Naloxone/Narcan to the person that overdosed during the last opioid overdose you witnessed? (Select one)
❑ Yes *(skip to #23c)* ❑ No ❑ Prefer not to say *(skip to #23c)*

23b. Why did you not give Naloxone/Narcan to the person experiencing an overdose? (Select all that apply)
❑ Some else gave Naloxone/Narcan ❑ Situation seemed under control
❑ Don't know how to use Naloxone/Narcan ❑ Naloxone/Narcan was not available
❑ Other, specify:____________________________________ ❑ Prefer not to say

23c. Was 9-1-1 called during the last opioid overdose you witnessed? (Select one)
❑ Yes *(skip to #24)* ❑ No ❑ Don't know *(skip to #24)* ❑ Prefer not to say *(skip to #24)*

23d. Why was 9-1-1 not called? (Select all that apply)
❑ Didn't have a phone/phone not available ❑ Situation seemed under control
❑ Worried about family services being notified
❑ Worried about neighbors/landlord knowing about drug use
❑ Worried about police coming. If so, specify why:_______________________________
❑ Other, specify:____
❑ Prefer not to say

24. Do you have a Naloxone/Narcan kit? (Select one)
❑ Yes ❑ No, I do not have a kit but I want one ❑ No, I don't have a kit and I don't want one ❑ Prefer not to say

25. In the __last 6 months__, did any of the following make it difficult for you to get a Naloxone/Narcan kit? (Select all that apply)
❑ Had no difficulties ❑ I don't need a kit ❑ The site where I can get a kit is too far away
❑ Worried about being stigmatized ❑ I don't know where to get a kit
❑ Other, Specify ____
❑ Prefer not to say

26. In the last 6 months, how often did you pick up supplies (e.g. needles) from any site/outreach, either for yourself or another person? (Select one)

❑ Every day ❑ A few times a week ❑ A few times a month ❑ Less than once a month
❑ Never ❑ Prefer not to say

27. In the last 6 months, did any of the following make it difficult for you to pick up supplies from any site/outreach? (Select all that apply)

❑ Had no difficulties ❑ Site not open ❑ Site too far away ❑ Staff had negative attitude
❑ Concerned about confidentiality ❑ Site didn't have the supplies I needed, specify:________________
❑ Other, specify: ________________________________ ❑ Prefer not to say

28. In the last 6 months, did any of the following make it difficult for you to dispose of used supplies at any site/outreach/drop box? (Select all that apply)

❑ Had no difficulties ❑ Not enough disposal locations nearby ❑ Disposal site hours were too short
❑ Worried about being stigmatized ❑ Other, specify: ____________________ ❑ Prefer not to say

29. In the last 6 months, have you injected any type of drug? (Select one)

❑ Yes ❑ No *(skip to #31)* ❑ Prefer not to say *(skip to #31)*

30a. In the last 6 months, did you have any trouble getting unused needles? (Select one)

❑ Yes ❑ No ❑ Prefer not to say

30b. In the last 6 months, have you fixed with a needle that had been used by someone else? (Select one)

❑ Yes ❑ No ❑ Prefer not to say

31. In the last 6 months, have you used drugs at an overdose prevention site (OPS)/supervised consumption site (SCS)? (Select one)

An OPS/ SCS is a place (fixed or mobile) where drug consumption is supervised by staff or volunteers to reduce overdose related deaths.

❑ Yes ❑ No *(Skip to #33)* ❑ Prefer not to say *(Skip to #33)*

32a. How often are you using an OPS/SCS? (Select one)

❑ Every day ❑ A few times a week ❑ A few times a month ❑ Less than once a month ❑ Prefer not to say

32b. In the last 6 months, what type of OPS/SCS have you used? (Select all that apply)

❑ Shelter or housing ❑ Community Health Centre/Health Clinic ❑ Stand-alone OPS/SCS facility
❑ Mobile Site ❑ Community Organization ❑ I wouldn't use a OPS/SCS
❑ Other, specify: ________________ ❑ Prefer not to say

33. In the last 6 months, did any of the following make it difficult for you to use an OPS/SCS? (Select all that apply)

❑ Had no difficulties ❑ I don't need to use an OPS/SCS ❑ Service not available nearby
❑ Concerned about confidentiality ❑ Worried about being stigmatized at OPS/SCS ❑ Staff had negative attitude
❑ Not allowed to smoke/snort drugs there ❑ Other, Specify: ________________________
❑ Prefer not to say

34. In the last 6 months, have you used a glass pipe (meth or crack) to smoke any drug? (Select all that apply)

❑ Yes, crack pipe ❑ Yes, meth pipe ❑ No, used something else to smoke (ie. light bulb, metal pipe)
❑ I don't use a pipe ❑ Prefer not to say

35. What do you do when you can't get a new (unused) pipe to smoke any drug? (Select all that apply)

❑ I don't use a pipe ❑ Inject instead ❑ Snort/swallow instead ❑ Wait until I find a new pipe
❑ Share, buy, or borrow a used pipe ❑ Smoke without a pipe using (specify): ________________
❑ I have never had a problem getting pipes ❑ Prefer not to say

36. In the last 6 months, did any of the following make it difficult for you to access Opioid Agonist Treatment (OAT)/Opioid Substitution Treatment (OST) (eg. methadone, buprenorphine/naloxone, etc)? (Select all that apply)

OAT/OST are drug therapies that counter opioid withdrawal symptoms and act as a substitute for the opioids you were previously taking

❑ Had no difficulties ❑ I did not try to access OAT/OST *(skip to #38)*
❑ I do not use opioids *(skip to #38)* ❑ Could not find a prescribing physician
❑ There were no pharmacies nearby ❑ Could not get prescription because of positive urine test
❑ Clinic fees were too high ❑ Worried about being stigmatized at clinic
❑ Wasn't offered preferred OAT/OST ❑ Other, specify: ________________________
❑ Prefer not to say

37a. In the last 6 months, were you prescribed any of the following OAT/OST? (Select all that apply)

❑ Methadone (Methadose) ❑ Buprenorphine/naloxone (Suboxone) ❑ Slow-release oral morphine (Kadian)
❑ Diacetylmorphine (heroin) ❑ Hydromorphone, pill form (generic) ❑ Hydromorphone, pill form (Dilaudid)
❑ Hydromorphone, injectable liquid (Dilaudid)
❑ Other, Please specify: ________________________________
❑ I wasn't prescribed any OAT/OST *(skip to #38)* ❑ Prefer not to say *(skip to #38)*

37b. In the last 6 months, did you discontinue OAT/OST? (Select one)

❑ Yes ❑No *(skip to #38)* ❑ Prefer not to say *(skip to #38)*

37c. Why did you discontinue OAT/OST? (select all that apply)

❑ Couldn't get to pharmacy during open hours ❑ Couldn't make clinic appointment time
❑ Treatment wasn't effective ❑ Switched treatment
❑ Clinic was too far away ❑ Challenges with transportation/travel
❑ Clinic staff had negative attitude ❑ Clinic fees were too high
❑ Prefer not to say ❑ Other, Please Specify:

38. Have you heard about the Good Samaritan Drug Overdose Act? (Select all that apply)

❑ Yes ❑ No *(skip to end)* ❑ Prefer not to say *(skip to end)*

39. Do you believe the GSDOA protects the following people from being arrested for simple possession of substances (small amount of drugs for own use) at the scene of an overdose? (Select all that apply)

a. The person who calls 9-1-1
   ❑ Yes ❑ No ❑ Prefer not to say
b. The person who overdoses
   ❑ Yes ❑ No ❑ Prefer not to say
c. Anyone at the scene of an overdose
   ❑ Yes ❑ No ❑ Prefer not to say

40. Imagine there is an overdose in a public place; 9-1-1 is called and the police come to the scene. Do you think the police can legally arrest a person if they: (Select all that apply)

a. Have a larger amount of drugs on them or items (eg. scale) that may look like they are involved in drug dealing
   ❑ Yes ❑ No ❑ Prefer not to say
b. Are in a red/no-go zone they received for a previous charge that was not simple drug possession (eg. theft)
   ❑ Yes ❑ No ❑ Prefer not to say
c. Have an outstanding warrant for something other than simple drug possession (eg. theft)
   ❑ Yes ❑ No ❑ Prefer not to say

Now I'd like to tell you about the ***Good Samaritan Drug Overdose Act****, which was made law in May 2017. It protects the person who overdoses, the person who calls 9-1-1, and anyone else at the scene of an overdose from being arrested for 'simple' possession that means having illegal drugs for their own personal use. It does not protect anyone at an overdose from being arrested for outstanding warrants, controlled substance trafficking or production, or any other serious offense. (Please provide the participant with a Good Samaritan Drug Overdose Act info card at this point.)*

Thank you for taking the survey!
