# Supplementary material for: Crystal methamphetamine use in British Columbia, Canada: A cross-sectional study of people who access harm reduction services
Source: PLoS One. 2021 May 26;16(5):e0252090. doi: 10.1371/journal.pone.0252090 (PMC8153500; doi:10.1371/journal.pone.0252090)
Supplement: S1 Table — (DOCX) [file pone.0252090.s003.docx]

| **S1 Table. Unadjusted and adjusted odds ratios and 95% confidence intervals for factors associated with past three-day self-reported crystal meth use (N = 1,107).** | | | |
| --- | --- | --- | --- |
| Characteristics^A^ | OR (95% CI) | AOR (95% CI) | *P*-value |
| **Study year** |  |  |  |
| 2018 | 1.00 | 1.00 | - |
| 2019 | 1.71 (1.33 – 2.20) | 1.76 (1.33 – 2.32) | < 0.001 |
| **Age** |  |  |  |
| <50 | 1.00 | 1.00 | - |
| ≥50 | 0.47 (0.35 – 0.62) | 0.60 (0.44 – 0.82) | 0.001 |
| Unknown | 0.41 (0.16 – 1.04) | 0.46 (0.15 – 1.38) | 0.164 |
| **Gender** |  |  |  |
| Man | 1.00 | 1.00 | - |
| Woman | 0.94 (0.71 – 1.22) | 0.96 (0.71 – 1.29) | 0.791 |
| Transgender and gender-expansive^B^ | 2.68 (0.77 – 9.30) | 2.84 (0.78 – 10.35) | 0.113 |
| Unknown | 1.76 (0.36 – 8.55) | 2.78 (0.50 – 15.43) | 0.242 |
| **Currently Regularly Housed** |  |  |  |
| Yes | 1.00 | 1.00 | - |
| No | 2.34 (1.74 – 3.14) | 2.11 (1.52 – 2.92) | < 0.001 |
| Unknown | 0.82 (0.40 – 1.68) | 0.93 (0.41 – 2.14) | 0.868 |
| **Currently Employed** |  |  |  |
| Yes | 1.00 | 1.00 | - |
| No | 1.88 (1.39 – 2.54) | 1.78 (1.27 – 2.50) | 0.001 |
| Unknown | 1.98 (0.99 – 3.98) | 2.06 (0.91 – 4.67) | 0.085 |
| **Opioid Use (L3D)**^C^ |  |  |  |
| Yes | 3.54 (2.72 – 4.59) | 3.28 (2.48 – 4.34) | < 0.001 |
| No | 1.00 | 1.00 |  |
| **Cannabis Use (L3D)** |  |  |  |
| Yes | 2.16 (1.67 – 2.79) | 2.19 (1.64 – 2.93) | < 0.001 |
| No | 1.00 | 1.00 |  |
| **Alcohol Use (L3D)** |  |  |  |
| Yes | 1.53 (1.17 – 2.00) | 1.47 (1.09 – 1.99) | 0.011 |
| No | 1.00 | 1.00 |  |
| Abbreviations: L3D, last three days; OR, odds ratio, AOR, adjusted odds ratio; CI, confidence interval  Unknown variable levels include missing responses and responses where participants indicated “prefer not to say”.  ^A^ All substances listed refer to self-reported use.  ^B^ Transgender and gender-expansive includes people that identified as transgender men, transgender women, and gender non-conforming.  ^C^ Opioids included heroin, fentanyl, morphine, oxycodone, and hydromorphone (Dilaudid) | | | |
